# Natural genetic variation determines susceptibility to aggregation or toxicity in a *C. elegans* model for polyglutamine disease

**DOI:** 10.1186/1741-7007-11-100

**Published:** 2013-09-30

**Authors:** Tali Gidalevitz, Ning Wang, Tanuja Deravaj, Jasmine Alexander-Floyd, Richard I Morimoto

**Affiliations:** 1 Department of Molecular Biosciences, Rice Institute for Biomedical Research, Northwestern University, Evanston, IL 60208-3500, USA; 2 Department of Biology, Drexel University, Papadakis Integrated Sciences Center Room 418, 3245 Chestnut Street, Philadelphia, PA 19104, USA; 3 Current address: Department of Natural Sciences, The University of Virginia’s College at Wise, Wise, VA 24293, USA

**Keywords:** Protein aggregation, Polyglutamine, Conformational disease, Genetic modifier, Natural variation

## Abstract

**Background:**

Monogenic gain-of-function protein aggregation diseases, including Huntington’s disease, exhibit substantial variability in age of onset, penetrance, and clinical symptoms, even between individuals with similar or identical mutations. This difference in phenotypic expression of proteotoxic mutations is proposed to be due, at least in part, to the variability in genetic background. To address this, we examined the role of natural variation in defining the susceptibility of genetically diverse individuals to protein aggregation and toxicity, using the *Caenorhabditis elegans* polyglutamine model.

**Results:**

Introgression of polyQ40 into three wild genetic backgrounds uncovered wide variation in onset of aggregation and corresponding toxicity, as well as alteration in the cell-specific susceptibility to aggregation. To further dissect these relationships, we established a panel of 21 recombinant inbred lines that showed a broad range of aggregation phenotypes, independent of differences in expression levels. We found that aggregation is a transgressive trait, and does not always correlate with measures of toxicity, such as early onset of muscle dysfunction, egg-laying deficits, or reduced lifespan. Moreover, distinct measures of proteotoxicity were independently modified by the genetic background.

**Conclusions:**

Resistance to protein aggregation and the ability to restrict its associated cellular dysfunction are independently controlled by the natural variation in genetic background, revealing important new considerations in the search for targets for therapeutic intervention in conformational diseases. Thus, our *C. elegans* model can serve as a powerful tool to dissect the contribution of natural variation to individual susceptibility to proteotoxicity.

Please see related commentary by Kaeberlein, http://www.biomedcentral.com/1741-7007/11/102.

## Background

A number of very serious human disorders are characterized by the appearance of protein aggregates, including neurodegenerative diseases such as Huntington’s disease (HD), metabolic disorders, and certain developmental disorders [1,2]. Hereditary forms of these diseases are caused by *de novo* gain-of-function mutations that result in expression of destabilized, aggregation-prone proteins [3,4], accompanied by the disruption of protein-folding homeostasis (proteostasis) and cellular dysfunction [5-11]. Although these mutations themselves are causative, many disease characteristics such as age of onset, penetrance, and severity of specific symptoms can vary widely between individuals. For example in HD, the age of neurological onset is strongly associated with the length of polyglutamine (polyQ) expansion in huntingtin protein. Yet age of onset can vary by several decades in people carrying the same length polyglutamine expansion, and a large proportion of this residual variation is genetic in nature and may be due to polymorphisms in other genes [12-15]. This variability is also seen in animal models of disease; for example, different genetic backgrounds of common laboratory mouse strains differentially modify somatic CAG repeat expansion and the onset of nuclear accumulation of mutant huntingtin in a knock-in HD mouse model [16-18]. It is generally well appreciated that genetic variation influences the phenotypic expression of mutations and transgenes, and the effects of environmental perturbations [19-23]. However, the nature of modifying alleles segregating in populations remains elusive for protein conformational diseases [15,24].

Much of the current knowledge about modifiers of conformational diseases has been gathered from linkage/association studies, candidate approaches, and unbiased genetic screens. The latter two approaches often use induced mutations, including knockouts/knockdowns and overexpression, in fixed genetic backgrounds. Although genetic variants identified in this way are enormously informative about pathways that affect disease processes and that can, when perturbed, modify disease phenotypes [25-30], they are likely to be different from the naturally occurring variations. First, these approaches are designed to identify individual modifier genes with strong phenotypic effects. By contrast, a naturally occurring genetic variation, in addition to the singleton genes with strong effect, is likely to be represented by complex networks of interactions of multiple common variants with small effects, and/or of rare or even private alleles with larger contributions [31-37], as well as cryptic genetic variation [38-40], thus making association of phenotypic differences with their underlying genotypes very challenging. Protein aggregation diseases present an additional unique challenge, owing to the sheer number of different processes and pathways that control proteostasis [30,41-43]. Modulation of proteostasis has been shown to buffer the expression of certain mutations and coding polymorphisms into phenotypes. For example, expression of aggregation-prone polyQ and SOD1 proteins [5,6], or down-modulation in plants and animals of activity of molecular chaperones, such as heat shock protein 90 (HSP90), unmasks buffered phenotypes caused by mildly destabilizing mutations segregating in populations [44-48], a phenomenon similar to the heat-induced phenocopies [49,50]. At the same time, the presence of such mild destabilizing variants in the genetic background modulates the aggregation and toxicity of aggregation-prone proteins, including polyQ [5,6]. An additional consideration in protein conformation diseases is the possibility of certain induced mutations, especially knockouts/knockdowns, to imbalance the proteostasis directly, for example by targeting a member of a multimeric complex and inducing a folding stress in the cell. Because imbalance in proteostasis can indirectly affect the aggregation phenotype of the disease-causing mutation, such genetic intervention may appear as a true modifier.

Secondly, because natural variants have been acted upon by selection, they are largely compatible with normal organismal development and function [23], in contrast to many of the modifiers of the induced-mutation type, which are not phenotypically innocuous. Moreover, genetic manipulations that protect cells and organisms from the toxic effects of protein aggregation can themselves be deleterious to the organism; many of the lifespan-extending mutations protect against proteotoxicity, but are incompatible with normal development, or are maladaptive under competitive conditions [51-53]. Similarly, overexpression of molecular chaperones or heat shock factor, which consistently protects against proteotoxicity, can be accompanied by slow growth and development, decreased fecundity, and aberrant signaling, and may even support transformed phenotypes [54-57]. Thus, identifying proteostasis modifiers from among natural variants shaped by selection may pinpoint the potential genes and networks that are naturally plastic [23] and thus can be pharmacologically modulated without negative effects on the organism.

In contrast to induced-mutation approaches, association studies do look at natural variants. However, the complexity of the genetic variation involved in control of proteostasis, as discussed above, makes these studies very difficult. Indeed, only a small number of genes have been firmly identified as modifiers of aggregation diseases by either candidate or association approaches [15,24]. Furthermore, these modifiers are often not replicated in different populations, and replicated modifiers account for only a small fraction of the heritable variation [15,58-60]. Genetic tractability of model animals, together with the ability to control the contribution of the environment, offers a unique opportunity to examine how natural variation in the genetic background of individuals affects their ability to resist protein aggregation; what is the nature of the genetic variants that modify proteostasis; whether these are distinct from the spectrum of induced mutations; and whether natural variation can predict/modify the susceptibility to protein conformation diseases.

Recombinant inbred lines (RILs) have been used successfully, from yeast to mice, to answer similar questions about non-transgenic physiological traits, including complex traits [18,61-66]. Here we established the polyglutamine transgenic *Caenorhabditis elegans* as one such model, taking advantage of the availability of genetically diverse wild isolates, and the ability to detect and measure protein aggregation in a live animal throughout its lifespan. In addition to the short generation time and lifespan of *C. elegans*, and the wealth of knowledge and reagents available for the pathways known to modulate aging and proteostasis, the facultative sexual reproduction mode of this organism allows for multifactorial perturbation of genetic variants through outcrossing, combined with the capture of true-breeding genes by self-reproduction, thus providing a rich resource for future mapping of the natural modifier alleles of protein aggregation and toxicity.

## Results

### Genetic background modulates age of onset and cell-specific susceptibility to polyQ aggregation

**Figure 1:**
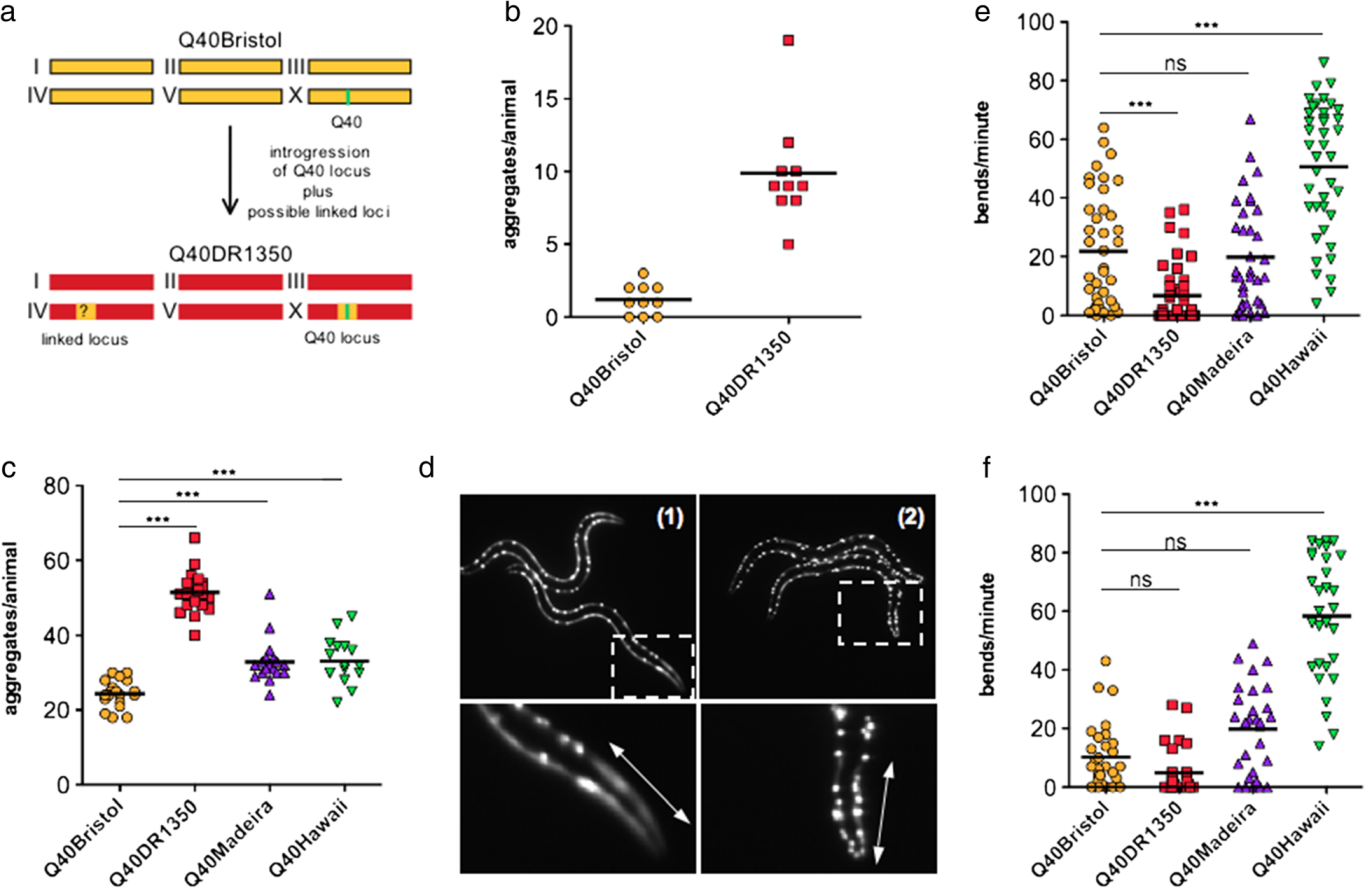
**Genetic background separately regulates polyQ aggregation and toxicity. (a)** Introgression of Q40 locus from Bristol to DR1350 background. The Q40 transgene is integrated on chromosome X. **(b)** Early onset of aggregation in DR1350 relative to Bristol background. Late L2 larvae were scored, 10 animals each. **(c)** Comparison of number of polyQ aggregates per L4 animal in different backgrounds. Each symbol represents an individual animal, at least 16 animals per genotype. **(d)** Distribution of polyQ aggregates in muscle cells in young adult (1) Q40Bristol and (2) Q40DR1350 animals. Lower panels show magnification of the selected regions, arrow indicates head muscles. **(e**,**f)** PolyQ-induced decrease in muscle function. Swimming motions (bends per minute) were scored for **(e)** day 2 or **(f)** day 3 adult animals of the indicated genotypes, 30 to 45 animals for each genotype. **(c**,**e**,**f)** Data were analyzed by ANOVA followed by Bonferroni’s multiple comparison test, α = 0.01, ***P<0.001. **(b)** Data were analyzed by unpaired *t*-test, two-tailed, with Welch’s correction, *P*<0.0001.

As described previously, expression of the near-threshold yellow fluorescent protein (YFP)-tagged polyglutamine expansion polyQ40-YFP (Q40) in muscle cells of *C. elegans* shows a striking age-dependent onset of aggregation, regulated by heat shock factor 1 (HSF1) and the insulin signaling pathway [25,26,67]. However, unlike the variability of polyQ-associated disease phenotypes in the human population, the behavioral, biochemical, and aggregation phenotypes in the *C. elegans* laboratory Bristol (N2) strain are homogeneous. To investigate whether natural genetic variation in *C. elegans* modifies polyQ aggregation and associated phenotypes, we introgressed the polyQ40 transgene from the Bristol background into three wild strains: a California-derived isolate DR1350, the Madeira isolate JU258, and a genetically distant Hawaii isolate CB4856 [68]. The introgression approach ensures that the transgene is present in the same copy number and genomic location in each of the resulting strains (Figure 1a), while long-term backcrossing (>35 generations) ensures a high degree of genetic homogeneity within each strain.

**Figure 2:**
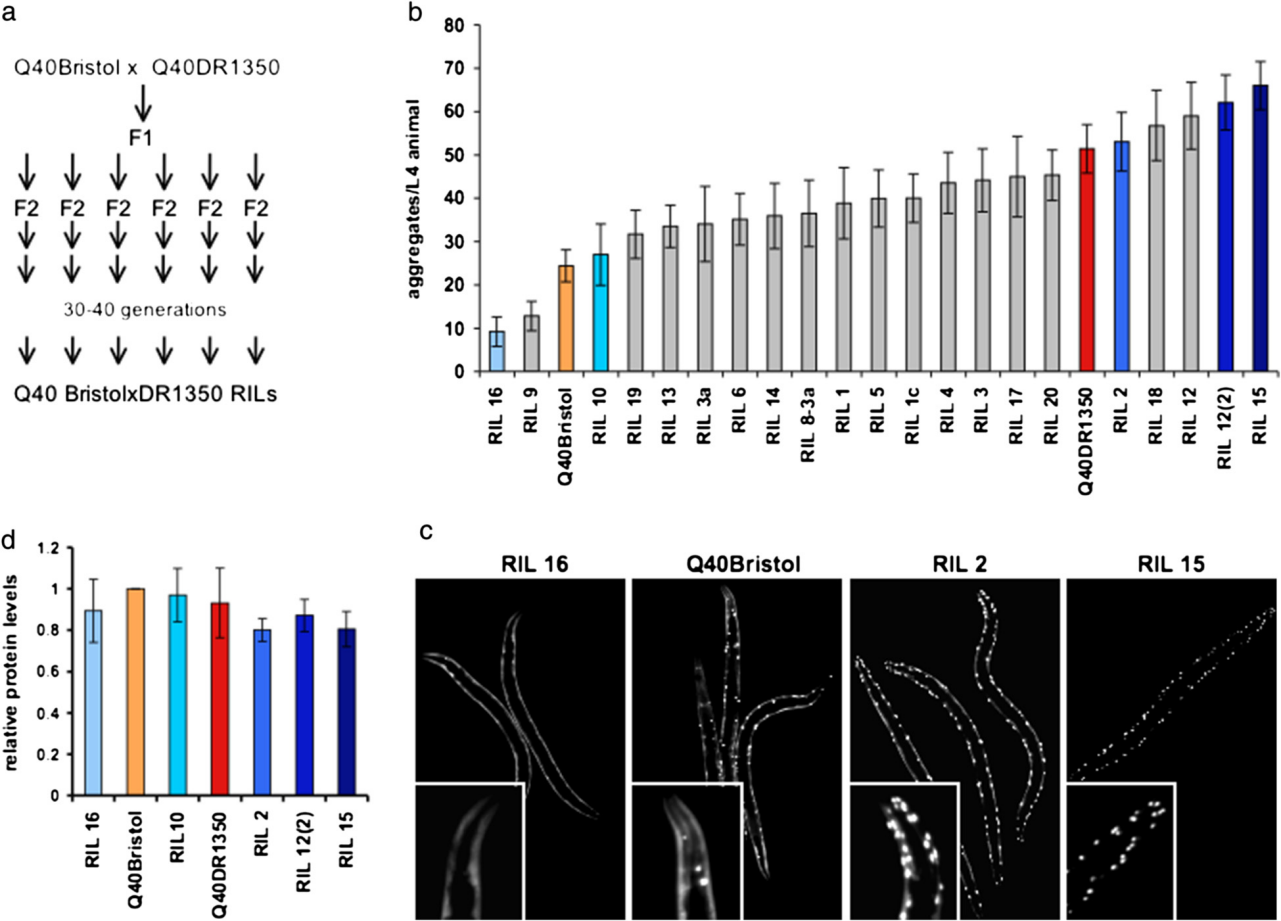
**Multiple alleles control polyQ aggregation in *Caenorhabditis elegans. *(a)** Scheme of generation of Q40Bristol × DR1350 recombinant inbred lines (RILs). To ensure genetic homogeneity within each RIL, 30 to 40 generations of self-reproduction were used. **(b)** Number of polyQ aggregates per L4 animal of parental strains and 21 RILs. Data are mean ± SD, 17 to 23 animals/data point. Colors correspond to selected strains as indicated in supplementary material (see Additional file 2: Figure S2a). **(c)** Images of representative animals used in **(b)**; insets show head regions. **(d)** Western blotting analysis of total Q40-YFP protein levels in extracts of L3 animals, relative to Q40Bristol. Data are mean ± SD, n = 3.

PolyQ aggregation and toxicity was strongly affected by introgression into wild strains. We observed an early onset of aggregation in DR1350 background, with on average eight-fold higher number of aggregates in the second larval stage (L2) of Q40DR1350 animals than in Q40Bristol animals (Figure 1b), and a twofold to threefold increase at the late larval stage 4 (L4) (Figure 1c, and data not shown). These early onset and increased aggregation were not due to any changes in polyQ40 expression (Figure 2d). By contrast, the Hawaii and Madeira backgrounds had only mild effects on polyQ aggregation (Figure 1c).

The relative susceptibility of subsets of muscle cells to polyQ aggregation was markedly different between the Bristol and DR1350 backgrounds. Head muscle cells were more resistant to aggregation than body wall muscles in Q40Bristol animals, whereas the inverse was true in the Q40DR1350 animals, with the head muscles exhibiting the earliest onset of polyQ aggregation. The head muscle cells of L2/L3 Q40DR1350 animals contained 74 ± 4% of all aggregates, compared with 22 ± 12% for Q40Bristol animals (mean ± SEM, n = 10). The Q40DR1350 strain retained this cell type-preferential aggregation phenotype throughout development (Figure 1d). The allele or alleles that confer increased susceptibility of this subset of muscle cells to aggregation appear to be unique to these two strains, as neither the Q40Hawaii nor Q40Madeira strains exhibited this phenotype.

### Genetic background differentially modulate polyQ aggregation and toxicity

Consistent with the earlier onset of aggregation, the Q40DR1350 strain exhibited an earlier onset of toxicity, with a twofold to threefold reduction in mean motility relative to Q40Bristol animals at days 1 to 3 of adulthood (Figure 1e,f; see Additional file 1: Figure S1a). This was in contrast to the Hawaiian background where this relationship was inverted, and reduced and delayed toxicity was apparent despite slightly increased aggregation. The Q40Hawaii strain had on average twofold improved motility relative to Q40Bristol on day 2 of adulthood, and this protection against polyQ toxicity was further enhanced to fivefold on day 3 of adulthood (Figure 1e,f). These results reveal that the Hawaiian background is highly protective against the toxic effects of polyQ40, without having any obvious effect on the visual appearance of aggregates (Figure 1c). This demonstrates that genetic polymorphisms that are present in the wild strains, and, therefore, acted upon by selection, are able to separately regulate the aggregation and resulting toxicity of polyglutamine protein. Thus, the genetic variants present in outbred populations provide natural protection against, or susceptibility to, protein aggregation and toxicity.

### Multiple alleles control polyQ aggregation in *C. elegans*

Because introgression of the polyQ40 transgene into different genetic backgrounds revealed the presence of natural variation in both aggregation and associated toxicity, we tested whether these phenotypes are controlled by the same or distinct genetic networks. To address this, we established a panel of RILs, each carrying the same polyQ40 transgene in the same genomic location, using the two backgrounds, that were most divergent for these phenotypes: Bristol and DR1350 (Figure 2a). Although each RIL carried a randomly generated assortment of parental alleles, the long-term inbreeding of each RIL ensured within-line homogeneity. This approach allowed for separation in the progeny of alleles that act epistatically or in concert in parental backgrounds, and for new configurations of genetic networks.

The 21 RILs exhibited a wide range of phenotypes from delayed onset and suppressed extent of aggregation in RILs 16 and 9, to strongly enhanced aggregation in RILs 12(2) and 15 (Figure 2b,c). RILs 15 and 16 showed transgressive behavior, with the number of aggregates in the majority of animals (88% for RIL 15 and 100% for RIL 16) being more than 2SD away from the mean of either of the parental lines, indicating contributions from multiple modifier alleles. The onset and extent of protein aggregation can be dependent on the expression levels, and we have previously reported delay in onset of aggregation in animals heterozygous for polyQ40 [5]. To address this, we measured polyQ-YFP fluorescence levels in extracts of L1 larva, prior to onset of aggregation, to determine whether it correlated with aggregation, and found no correlation between expression levels and aggregation of polyQ protein in RILs (R^2^ = 0.055) (see Additional file 2: Figure S2a).

We further selected several RILs with similar expression levels, which were either most suppressed or most enhanced in terms of aggregation, and two RILs whose aggregation was similar to each of the parental lines (Figure 2b, colored bars; see Additional file 2: Figure S2b), resulting in five RILs and two parental lines binned into four aggregation groups. Similar polyQ expression levels in these strains were confirmed by measuring total polyQ-YFP protein levels in L3 animals by western blotting analysis, showing that neither enhancement (RILs 12(2) and 15), nor suppression (RIL 16) of aggregation in these selected lines was due to a change in expression levels of polyQ protein (Figure 2d). Furthermore, increased (RIL 2) or decreased (RIL 16) aggregation as detected by visual appearance of fluorescent foci in day 1 adult animals correlated with increased or decreased levels of polyQ protein in the SDS-resistant high molecular weight fraction (see Additional file 2: Figure S2c). Thus, polyQ aggregation appears to be a transgressive trait, with some RIL progeny occupying phenotypic extremes relative to the parental strains (Figure 2b), indicating that multiple alleles with opposing or epistatic effects control protein aggregation.

### Natural variation independently controls resistance to aggregation and the ability to restrict its associated cellular dysfunction

In both parental lines, we found expression of the aggregation-prone polyQ40 to be associated with muscle toxicity (Figure 1e,f; see Additional file 1: Figure S1). Thus, we investigated whether natural variants segregating in the RIL progeny population were coordinately regulating polyQ aggregation and toxicity. We reasoned that RILs with enhanced aggregation would show earlier or more severe organismal correlates of polyQ toxicity, and *vice versa*. To monitor toxicity phenotypes, we scored motility and egg-laying, traits that directly report on the dysfunction of two different muscle subtypes, namely, body wall and reproductive muscles. We also scored shortening of the lifespan, which is a more indirect measure of organismal dysfunction caused by expression of polyQ40 in the muscle cells. The ability to simultaneously score these different toxicity measures in the same animals allows comparison of cell type-selective and global consequences of polyQ toxicity.

**Figure 3:**
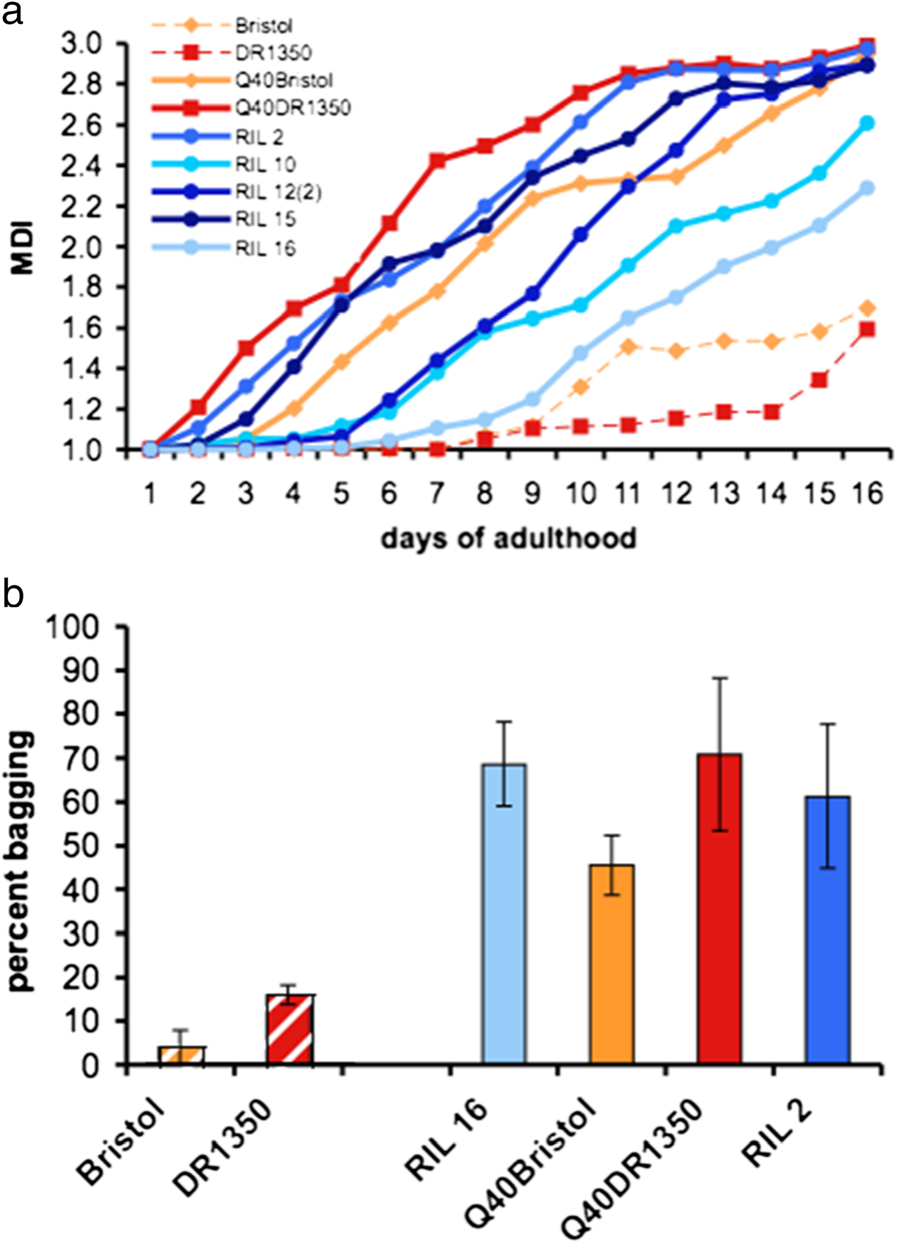
**Large variation in toxicity correlates. (a)** Progressive increase in muscle dysfunction index (MDI) during adulthood and aging of indicated strains: MDI is 1 when all animals on the plate move spontaneously, and MDI is 3 when all are paralyzed. The curves are a rolling average with a period of 2 days. **(b)** Internal hatching (bagging) phenotype due to dysfunction in reproductive muscles, scored over the entire egg-laying period. Data are mean ± SD, at least 30 animals per trial, n = 3 trials.

**Figure 4:**
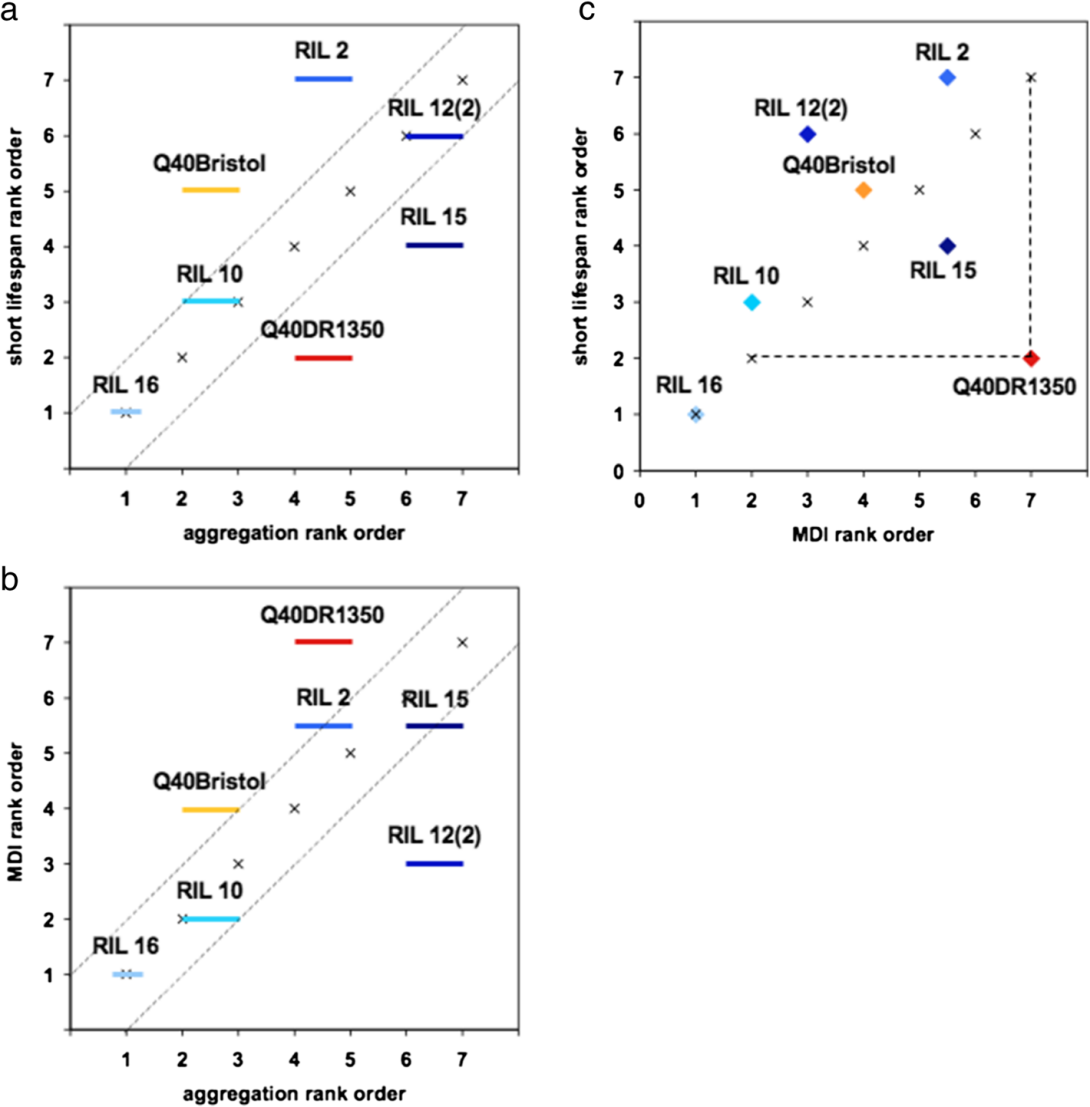
**Genetic background determines resistance and tolerance of polyQ aggregation. (a,b)** Relative ranking of indicated polyQ-carrying strains in aggregation and two toxicity correlates: **(a)** abnormally short lifespan and **(b)** muscle dysfunction. Aggregation ranking is as described in supplementary materials (see Additional file 2: Figure S2b). Strains that are paired share aggregation rank, and RIL 16 is unpaired. Placement above or below the diagonal indicates decreased or increased tolerance, respectively. **(c)** Variation in polyQ aggregation tolerance measured by shortening of lifespan could be separated from that measured by muscle dysfunction. DR1350 is indicated as an example of decreased tolerance in terms of muscle dysfunction relative to lifespan.

Motility was measured by adapting a population-level muscle dysfunction index (MDI; see Methods), that reports on the gradual deterioration of movement over the lifespan [69] (Figure 3a). We found that the MDI overall tracked with enhanced or suppressed aggregation for the five RILs scored (Figures 2b, 3a, 4b). For example, RIL 16 exhibited the lowest level of aggregation (ranked 1 for aggregation, see Additional file 2: Figure S2b) and was the most protected of the polyQ40-containing lines (ranked 1 for MDI, Figures 3a and 4b), whereas RIL 15 had early onset of both aggregation and muscle dysfunction. However, there was not an absolute correspondence in relative ranking: RIL 12(2), representing the most enhanced aggregation bin (ranked 6 to 7, see Additional file 2: Figure S2b), had a similar time to onset of muscle dysfunction as the much less aggregating RIL 10 (Figures 3a, 4b), indicating that variants present in RIL 12(2) protected muscle cells from the toxic effects of polyQ aggregation, but not from the protein aggregation itself. Two of the RILs examined, RILs 16 and 10, exhibited a phenotype intermediate between the non-transgenic Bristol and DR1350 strains and the parental Q40Bristol and Q40DR1350 strains (Figure 3a). Thus, polyglutamine aggregation and the associated muscle dysfunction are both controlled by complex but non-overlapping allelic variation present in *C. elegans* strains.

Shortening of lifespan also showed incomplete correspondence with enhanced or suppressed aggregation. The reduced aggregation RILs 16 and 10 had the longest lifespan (approximately 18.6 and 17 days, respectively) of the seven strains scored (Table 1; Figure 4a). However, the highest aggregating RIL 15 had only intermediate shortening of the lifespan. Furthermore, although Q40DR1350 and RIL 2 belonged to the same aggregation bin (see Additional file 2: Figure S2b), they had very disparate shortening of the lifespan (approximately 17 and 13.5 days, respectively) (Table 1), with one having a shorter and one a longer lifespan than that predicted by the aggregation rank (Figure 4a).

**Table 1:** Lifespan analysis

**Name**	**Number**	**Restricted mean**			**Age in days at percentage mortality**					**95% median CI**
		**Days**	**SEM**	**95% CI**	**25%**	**50%**	**75%**	**90%**	**100%**	
daf-16	119	16.17	0.51	15.16 to 17.18	13	15	19	22	31	14 to 15
age-1	107	29.57	1.62	26.39 to 32.75	17	27	42	50	53	22 to 30
Bristol	100	19.92	0.84	18.26 to 21.57	13	21	25	29	31	19 to 21
DR1350	123	22.75	0.85	21.09 to 24.41	21	23	26	28	34	21 to 25
Q40Bristol	120	14.89	0.46	13.98 to 15.80	11	15	17	20	28	15 to 15
Q40DR1350	102	18.32	1.06	16.24 to 20.39	12	18	25	26	28	15 to 22
Q40DR1350	40	16.53	0.99	14.59 to 18.48	12	17	22	23	24	15 to 19
RIL 2	100	13.6	0.54	12.54 to 14.67	12	13	16	19	21	13 to 14
RIL 2	102	13.49	0.49	12.53 to 14.45	11	13	17	18	26	12 to 12
RIL 10	117	17.33	0.51	16.33 to 18.34	15	17	20	23	28	15 to 17
RIL 12(2)	100	13.5	0.59	12.35 to 14.64	10	14	18	19	23	12 to 14
RIL 12(2)	116	14.66	0.34	13.99 to 15.33	12	15	17	19	26	14 to 14
RIL 15	70	15.69	0.91	13.90 to 17.48	12	15	18	21	26	14 to 17
RIL 15	111	14.81	0.68	13.49 to 16.14	12	15	17	21	33	12 to 14
RIL 16	91	18.6	1.11	16.43 to 20.76	14	19	23	25	26	15 to 22
RIL 16	115	18.65	0.99	16.70 to 20.60	15	17	23	28	27	15 to 19

RIL, recombinant inbred line.

Because progressive muscle dysfunction in *C. elegans* has been linked to aging [69], we compared the lifespan and MDI ranking of the same animals, and found that polyQ toxicity measured by muscle dysfunction could be uncoupled from the shortening of the lifespan. For example, relative to its aggregation rank, Q40DR1350 had a high degree of muscle dysfunction (ranked seventh out of seven in MDI; Figure 3a), but only mild shortening of lifespan (approximately 17 days, second out of seven (Figure 4c; Table 1). Overall, these data showed that in some genetic backgrounds there is correspondence between aggregation, cellular dysfunction, and lifespan, whereas other backgrounds appear to contain non-overlapping variation that uncouples aggregation from one or both measures of toxicity.

Finally, we scored an internal hatching phenotype (‘bag of worms’; *bag*) that reports on the function of reproductive muscle cells (Figure 3b). Unexpectedly, we found that the expression of polyQ40 caused significant toxicity in the reproductive tract, irrespective of the overall aggregation status (for example, compare RIL 16 and RIL 2; Figures 2b and 3b). The *bag* phenotype was undetectable or was only at background levels in the first 3 days of the reproduction period, suggesting that it was degenerative in nature and not due to a defect in muscle development. Thus, it appears that not only it is possible to uncouple aggregation and toxicity through the influence of natural genetic variation in *C. elegans*, but there are also intrinsic differences in the relative sensitivity of muscle cell types to the expression of polyglutamine expansion.

## Discussion

Our studies establish *C. elegans* as a potent model to probe the natural variation that shapes the susceptibility of individual animals to the toxic effects of protein aggregation. We found that three of the wild backgrounds used in this study had modifying effects on multiple aspects of polyglutamine aggregation and toxicity, including the onset and extent of aggregation, the differential susceptibility of specific subsets of muscle cells to aggregation, and the direct cellular and indirect organismal correlates of dysfunction. Another important finding in this model is that both polyQ aggregation and toxicity behaved as complex traits, suggesting that multiple additive or interacting alleles from wild *C. elegans* backgrounds are contributing to this phenotypic variation. Thus, this model will provide an invaluable tool to investigate the nature of alleles that contribute to the robustness of proteostasis in genetically diverse but phenotypically normal individuals.

Much progress has been made in understanding the mechanisms by which protein misfolding and aggregation causes cellular toxicity, and the pathways and networks that can be protective [30,41,43,70-72]. However, because the common candidate approaches (mutational, knockout/RNA interference (RNAi), or overexpression) are designed to detect genes that individually have large phenotypic effects, they often identify modifier genes that are not easy targets for intervention. Many of the genes identified as important for maintenance of proteostasis, including molecular chaperones, regulators of stress responses, insulin signaling, and autophagy, are themselves either essential, or have additional strong effects on development and/or normal physiology [54,55,57]. Nevertheless, modifier backgrounds do exist in the outbred human population, as evidenced by the large variability in age of onset or even penetrance of protein misfolding diseases. This suggests that natural genetic variants that are shaped by selection can both provide protection against protein aggregation and toxicity, and, at the same time, be compatible with normal development and physiology. Thus, natural variation may tag the genetic and molecular pathways whose inherent plasticity allows for development of intervention strategies without the negative or toxic effects.

Previous mutational studies have shown that protein aggregation can be uncoupled from toxicity [73]. For example, decreased insulin signaling causes increased aggregation but decreased toxicity of Aβ in *C. elegans*[74], whereas overexpression of HSP70 rescues polyQ toxicity in *Drosophila* by affecting the properties but not the extent of aggregation [75,76]. These studies suggest that changing the aggregation pathway may translate into changes in toxicity; however, the question remains whether the two are always related causally, or whether they are controlled by molecular pathways that can be modified independently of each other. Studies of plant-pathogen and animal-pathogen interactions show that resistance (ability to limit pathogen burden) and tolerance (ability to limit fitness loss at a given pathogen load) can have distinct genetic bases, and that genetic variation exists for both [77,78]. We suggest that a similar logic could be applied to proteotoxic mutations. We found that identical genetic lesions (the same transgene introgressed into different strains) produced vastly different aggregation phenotypes, supporting variation in resistance to aggregation, including at the level of cell type-specific resistance in head versus body wall muscle cells. At the same time, strains that exhibited similar levels of polyQ aggregation differed widely in toxicity correlates, such as muscle dysfunction and shortening of lifespan. Although further research will be needed to characterize these phenomena, these findings indicate a remarkable variation at the organismal level in the ability to tolerate protein aggregation. This is clearly evident in natural isolates of *C. elegans*, as the Hawaiian strain showed increased tolerance of polyQ aggregation as measured by the loss of motility, whereas DR1350 showed both decreased resistance and decreased tolerance in terms of muscle dysfunction, but increased tolerance when measured by the shortening of lifespan. Furthermore, it appears that natural variants contributing to resistance and tolerance can be found even within the limited RIL panel used in this study; relative to the parental strains, RIL 16 demonstrated remarkable resistance and tolerance, the latter in both of the phenotypic measures for which variation was present.

Our data thus reveal that distinct genetic networks control protein aggregation and the various cellular and organismal consequences of such aggregation. One potential scenario for this distinction is that pathways that affect resistance to aggregation act directly on the mutant aggregation-prone protein, for example, by changing its folding trajectory or degradation rate, whereas those that affect tolerance of protein aggregates also affect specific cellular pathways that are disrupted in disease, or affect parallel or compensatory pathways. This suggests that, depending on the individual’s genetic background, disease intervention strategies may need to distinguish between the strategy of prevention of misfolding and aggregation from an alternative strategy of strengthening specific cellular pathways that mediate disease phenotypes.

## Conclusions

These results establish *C. elegans* as a potent model to study how natural variation shapes the susceptibility to proteotoxicity in genetically diverse but phenotypically normal individuals, and support the view that intrinsic aggregation propensity of the mutant disease-related proteins is not, by itself, sufficient to explain disease onset and phenotypes. We found that both protein aggregation and toxicity behave as complex traits, suggesting that multiple additive or interacting alleles contribute to the variation in these phenotypes. The ability of standing genetic variation in wild isolates of *C. elegans* to independently modify the age of onset, the cell type-specific susceptibility to protein aggregation, and several independent toxicity correlates of the polyglutamine expansion, suggests not only multiple paths to the expression of toxicity, but also multiple potential routes of intervention. Because natural variation represents genetic and molecular pathways that are inherently plastic, identifying these pathways will allows for development of intervention strategies that avoid the negative or toxic effects.

## Methods

### Nematode strains and growth conditions

Nematodes were grown at 20°C on nematode growth medium (NGM) plates seeded with *Escherichia. coli* OP50 [79]. Animals were synchronized by picking the pre-comma stage embryos onto fresh plates, or by hypochlorite treatment. Wild *C. elegans* strains were obtained from the Caenorhabditis Genetic Center. The Q40Bristol strain is AM737. To introgress Q40-YFP transgene into the wild backgrounds, Q40Bristol males were mated with DR1350, JU258(Madeira), or CB4856(Hawaii) hermaphrodites, then five to seven F1 fluorescent hermaphrodites were mated with wild males, and this two-step cycle was repeated until over 35 generations of backcrosses to the wild background were reached. At this point, strains were homozygosed for polyQ40 and cryopreserved. To generate RILs, fluorescent F2 animals from a cross between Q40Bristol males and DR1350 hermaphrodites were singled out and allowed to self-reproduce for 30 to 40 generations, by transferring 25 to 30 L4 hermaphrodites to new plates per generation. Strains were cryopreserved every 10 to 15 generations, and homozygosed for Q40 after generation 10. For RNAi experiments, nematodes were cultured on control (L4440 empty vector, GeneService, USA) or YFP RNAi plates from early embryo stage. NGM plates containing 100 μg/ml ampicillin, 0.4 mol/l isopropyl β-D-1-thiogalactopyranoside (IPTG) and 12 μg/ml tetracycline (Sigma) were spotted with overnight RNAi bacterial cultures, and incubated for 2 days prior to embryo plating. YFP RNAi resulted in a strong decrease in polyQ-YFP levels in Q40Bristol, and a weaker decrease in Q40DR1350.

### PolyQ aggregation

Except for one experiment (shown in Figure 2b), the number of aggregates per animal was scored by counting fluorescent foci in live animals, using stereomicroscopes (MZ16FA or M205FA; Leica Microsystems, Switzerland). Correct developmental stages were verified based on morphological features. For allele assessment (Figure 2b,c), nematodes were mounted on slides, and images were taken using an Axiovert 200 microscope (Carl Zeiss, Jena, Germany) with a digital camera (C4742-98; Hamamatsu). Numbers of aggregates were then quantified using a custom plugin (Dr Jesper S. Pedersen; unpublished data) for ImageJ software (National Institutes of Health). Aggregates in the head muscles were counted above the border between pharynx and intestinal valve.

To determine transgression, we compared the aggregation range of the outlier RILs to the parental strains. All the scored RIL 16 animals were in 4 to 16 aggregates per animal range (see Additional file 2: Figure S2b), more than 2SD away from the mean of the less aggregated parent, Q40Bristol (mean ± SD 24.4 ± 3.7). Conversely, 88% of scored RIL 15 animals were in 63 to 80 aggregates per animal range, more than 2SD away from the mean of the more aggregated parent, Q40DR1350 (51.5 ± 5.4=).

To measure the ratio between aggregated and soluble polyQ40-YFP protein, native extracts were prepared as described previously [6], incubated in 5% SDS at room temperature, and resolved by non-denaturing PAGE. Protein was visualized and quantified on Storm860 scanner (Molecular Dynamics, USA) equipped with a blue laser.

PolyQ-YFP protein levels in L1 animals, prior to the onset of aggregation, was determined by measuring YFP fluorescence, normalized to the total protein concentration (bicinchoninic acid assay; Pierce, Rockford, IL, USA). Briefly, embryos were collected by hypochlorite treatment, hatched overnight in the absence of food to ensure synchronization, and frozen at −80°C in aliquots. Lysates were prepared by brief sonication followed by centrifugation at 14,000 × *g*. Expression levels of Q40-YFP in L1 animals of 20 RILs and two parental lines were within 50% of each other.

Total polyQ40-YFP levels were quantified in L3 larval animals by western blotting with goat anti-GFP IRDye800 antibody (Rockland Immunochemicals, USA) using Odyssey scanner (LI-COR, USA), in samples prepared by boiling a known number of L3 larval animals in 3% SDS. YFP levels were normalized to α-tubulin with anti-α-tubulin primary antibody (Sigma,) followed by Alexa Fluor 680 goat anti-mouse IgG (Molecular Probes, USA). We chose L3 larvae in order to minimize the effect of different turnover rates between aggregated and soluble proteins between lines with suppressed and enhanced aggregation, and boiling in SDS ensured complete dissolution of aggregates.

### Correlates of toxicity

#### Motility

To measure the number of bends per minute in swimming animals, individual nematodes were picked into a drop of M9 buffer, allowed to acclimate for 1 minute, and then complete bends were counted for 1 minute. A complete bend was considered to occur when both the anterior and posterior of the animal crossed the vertical axis twice.

#### MDI

To measure MDI, we modified the approach of Herndon *et al.*[69] to provide a population-level measure of the gradual decline in the muscle function of the same group of animals over time. MDI and lifespan (see below) were measured simultaneously in the same groups of animals. Briefly, individual animals were given a score of 1 if they moved unprovoked or moved rapidly after a gentle touch with a pick or exposure to 1 to 2 seconds of blue light; a score of 2 if they either moved slowly when unprovoked or could move their entire body after gentle stimulation; and a score of 3 if they were completely paralyzed or could move only the head but not the body. The scores were averaged over the number of animals tested to produce the MDI. At the extremes, MDI = 1 indicate that all animals on the plate can move well, whereas MDI = 3 indicates that all animals are paralyzed.

#### Internal hatching (bagging)

Animals were examined for internal hatching for the duration of their reproductive period. In many cases, one or only a few embryos were retained at the end of reproduction, resulting in internal hatching but not the typical ‘bag of worms’ appearance. One of the three trials was performed simultaneously with lifespan/MDI on the same group of animals.

#### Lifespan

Animals were cultured on OP50 bacteria without fluorouracil deoxyribose (FUdR), and transferred away from their progeny every other day during reproductive period. Animals were examined for signs of life initially daily and then every 2 to 3 days by observing their movement. Animals that were not moving or twitching after gentle stimulation, followed by vigorous stimulation, or that did not exhibit pharyngeal pumping for 30 seconds, were considered dead. Animals that had internal hatching, or that crawled off the plates, were censored. Data analysis was performed as described by Yang *et al*. [80], using the publicly available analysis suite Oasis [81].

### Statistics

All ANOVA and *t*-test analyses were performed using Prism software (GraphPad, USA). ANOVA was followed by multiple comparison post-test, either between selected pairs, or between all groups, as indicated in the figures, and α and significance levels are also indicated in the figures.

## Competing interests

The authors declare that they have no competing interests.

## Authors’ contributions

RIM and TG designed the experiments; TG, NW, TD, and JAF performed the experiments; TG performed the statistical analysis; and RIM and TG wrote the manuscript. All authors read and approved the final manuscript.

## Supplementary Material

Additional file 1: Figure S1 Muscle dysfunction is due to polyQ expression. **(a,b)** Swimming motions (bends per minute) were scored in **(a)** day 1 or **(b)** day 2 adult Q40Bristol and Q40DR1350 animals grown on control (empty vector) or yellow fluorescent protein (YFP) RNA interference (RNAi). YFP RNAi decreases expression of Q40-YFP fusion protein, resulting in improvement in motility. YFP RNAi was partially effective in a DR1350 background. **(a)** Q40Bristol animals had little loss of motility on control plates at day 1, but had **(b)** a significant loss of motility on day 2. **(c)** Motility of the non-transgenic parental strains, at least 15 animals per genotype. **(a, b)** Independent experiments, 15 and 10 animals per treatment, respectively. **(a, c**) Data were analyzed by ANOVA followed by Bonferroni’s multiple comparison test, α = 0.01, **P>0.001 and <0.01, ***P<0.001. **(b)** Data were analyzed by unpaired *t*-test, two-tailed, *P*<0.0001. Click here for file

Additional file 2: Figure S2 Strains could be ranked according to polyQ aggregation. **(a)** Lack of correlation between polyQ-YFP expression levels in L1 animals and its aggregation across the recombinant inbred lines (RILs). Aggregation data are the same as in Figure 2b. Data points in color correspond to the selected RILs and parental strains as in panel **(b)**. **(b**) Statistical analysis of polyQ aggregation in selected strains. Each symbol represents an individual animal, and data are the same as in Figure 2b. RIL 16 represents a suppressed aggregation phenotype, ranked as 1 for aggregation; RIL 10 is similar to Q40Bristol, both ranked as 2 to 3; RIL 2 is similar to Q40DR1350, ranked as 4 to 5; and RILs 12(2) and 15 represent an enhanced aggregation phenotype, ranked as 6 to 7. Data were analyzed by ANOVA followed by Bonferroni’s multiple comparison test (all pair combinations), α = 0.01. The indicated pairs of strains are not significantly different from each other, and all other combinations are *P*<0.001. **(c)** Ratio of polyQ-yellow fluroescent protein (YFP) in SDS-resistant high molecular weight and soluble monomeric species. Click here for file
