# Supplementary figures and images for: Natural genetic variation determines susceptibility to aggregation or toxicity in a C. elegans model for polyglutamine disease
Source: BMC Biol. 2013 Sep 30;11:100. doi: 10.1186/1741-7007-11-100 (PMC3816611; doi:10.1186/1741-7007-11-100)

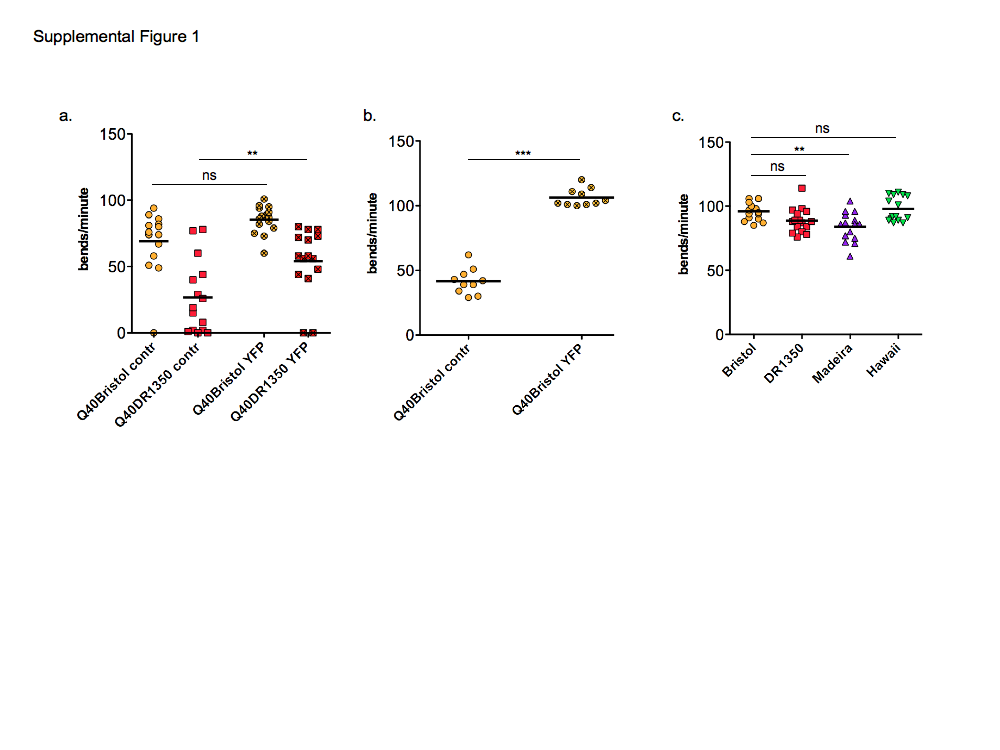

Supplement: Additional file 1: Figure S1 — Muscle dysfunction is due to polyQ expression. (a,b) Swimming motions (bends per minute) were scored in (a) day 1 or (b) day 2 adult Q40Bristol and Q40DR1350 animals grown on control (empty vector) or yellow fluorescent protein (YFP) RNA interference (RNAi). YFP RNAi decreases expression of Q40-YFP fusion protein, resulting in improvement in motility. YFP RNAi was partially effective in a DR1350 background. (a) Q40Bristol animals had little loss of motility on control plates at day 1, but had (b) a significant loss of motility on day 2. (c) Motility of the non-transgenic parental strains, at least 15 animals per genotype. (a, b) Independent experiments, 15 and 10 animals per treatment, respectively. (a, c) Data were analyzed by ANOVA followed by Bonferroni’s multiple comparison test, α = 0.01, **P>0.001 and <0.01, ***P<0.001. (b) Data were analyzed by unpaired t-test, two-tailed, P<0.0001. [file 1741-7007-11-100-S1.tiff]

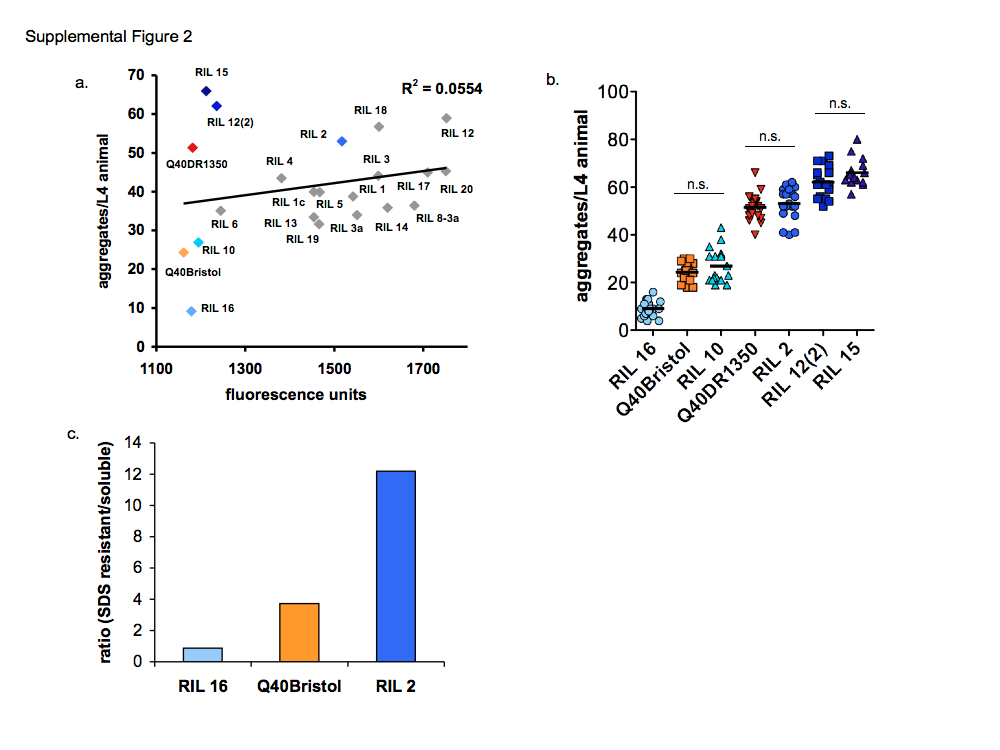

Supplement: Additional file 2: Figure S2 — Strains could be ranked according to polyQ aggregation. (a) Lack of correlation between polyQ-YFP expression levels in L1 animals and its aggregation across the recombinant inbred lines (RILs). Aggregation data are the same as in Figure 2b. Data points in color correspond to the selected RILs and parental strains as in panel (b). (b) Statistical analysis of polyQ aggregation in selected strains. Each symbol represents an individual animal, and data are the same as in Figure 2b. RIL 16 represents a suppressed aggregation phenotype, ranked as 1 for aggregation; RIL 10 is similar to Q40Bristol, both ranked as 2 to 3; RIL 2 is similar to Q40DR1350, ranked as 4 to 5; and RILs 12(2) and 15 represent an enhanced aggregation phenotype, ranked as 6 to 7. Data were analyzed by ANOVA followed by Bonferroni’s multiple comparison test (all pair combinations), α = 0.01. The indicated pairs of strains are not significantly different from each other, and all other combinations are P<0.001. (c) Ratio of polyQ-yellow fluroescent protein (YFP) in SDS-resistant high molecular weight and soluble monomeric species. [file 1741-7007-11-100-S2.tiff]
